# Associations between the nutritional quality of snacks, overall diet quality and adiposity: findings from a nationally representative study of Australian adolescents

**DOI:** 10.1017/S0007114524001727

**Published:** 2024-08-28

**Authors:** Binyam Girma Sisay, Sarah A. McNaughton, Kathleen E. Lacy, Rebecca M. Leech

**Affiliations:** 1 Institute for Physical Activity and Nutrition, School of Exercise and Nutrition Sciences, Deakin University, Melbourne, VIC 3125, Australia; 2 School of Exercise and Nutrition Sciences, Deakin University, Melbourne, VIC, Australia; 3 Health and Well-Being Centre for Research Innovation, School of Human Movement and Nutrition Sciences, University of Queensland, St Lucia, QLD 4067, Australia

**Keywords:** Snacks, Snack nutritional quality, Adiposity, Diet quality, Adolescents

## Abstract

The primary aim of this study was to examine the association between snack nutritional quality, overall diet quality and adiposity among Australian adolescents. The secondary aim was to assess the distribution of discretionary foods (i.e. energy-dense and nutrient-poor foods and beverages) and intakes from the five food groups at different levels of snack nutritional quality. Dietary data collected from nationally representative adolescents (12–18 years old) during a 24-h dietary recall in the National Nutrition and Physical Activity Survey were analysed (*n* 784). Snacks were defined based on participant-identified eating occasions. Snack nutritional quality was assessed using the Nutrient Profiling Scoring Criterion (NPSC), whereas diet quality was evaluated using the Dietary Guideline Index for Children and Adolescents. Adiposity was assessed through BMI Z-score waist circumference and waist:height ratio (WHtR). Higher nutritional quality of snacks, as assessed by the NPSC, has been associated with higher diet quality among both boys and girls (*P* < 0·001). However, there is no association between snacks nutritional quality with BMI Z-score, waist circumference and WHtR. Among both boys and girls, the consumption of fruits, vegetables and legumes/beans at snacks increased with improvement in snack nutritional quality. Conversely, the consumption of discretionary foods at snack decreased with improvement in snack nutritional quality. In conclusion, improved snack quality was associated with better diet quality in adolescents. However, there was no association between snack nutritional quality and adiposity. Future, snack nutrition quality indices should consider optimum snack characteristics related with adiposity and diet quality.

Dietary patterns (i.e. fruits, vegetables and sugary foods) developed during adolescence often continue into adulthood^(1–3)^. Poor diet quality during adolescence, which is characterised by a high intake of discretionary foods (i.e. energy-dense and nutrient-poor foods and beverages) and low intake of foods from the five food groups (i.e. grain (cereal) foods, vegetables and legumes/beans, fruit, milk, yoghurt, cheese and/or other alternatives, lean meats and poultry, fish, eggs, tofu, nuts and seeds and legumes/beans). Poor diet quality among adolescents is associated with obesity and cardiometabolic risks, such as insulin resistance and elevated blood pressure, which can persist into adulthood^(4–6)^. Therefore, investigating dietary behaviours that can enhance diet quality and address the current burden of obesity and related diseases is a crucial public health priority.

To date, nutritional research in adolescents (10–19 years) has primarily examined the impact of nutrient and energy intake on obesity and related diseases^(7–9)^. However, foods are typically consumed in combination at eating occasions, including meals or snacks, and these combinations may have interactive or synergistic effects on health^(10)^. Snacking (i.e. eating occasion between meals) is a common dietary behaviour, constituting more than a quarter of adolescents’ total energy intake^(11)^. From 1997 to 2014, the percentage of USA adolescents who snacked increased from 61 % to 83 %, and the energetic content of snacks also increased from 307 kcal to 461 kcal per snack occasion^(12)^. This increase in the prevalence of snacking accompanied by increasing energy content per snack highlights the need to understand the role of snacks in adolescent diet quality. Only a few studies have examined the association between snacks, diet quality and obesity among adolescents and the findings are conflicting^(13–17)^. The lack of clear evidence around snacks is reflected in the dearth of national food-based dietary recommendations for adolescents on snack consumption.

Studies examining the relationship between snacking, diet quality and obesity have mostly examined the frequency of snack consumption within a day, the contribution of snacks to daily energy intake and the energy density (ED) of snacks^(13–15,18,19)^. Among these characteristics, both snack frequency and energy intake from snacks are unlikely to capture the nutritional content or quality of snacks and may explain the conflicting findings on the associations between snacking, diet quality and obesity. In contrast, ED may be a more informative characteristic of snacks due to its relationship with adiposity^(20)^ and the tendency for lower ED diets to be associated with higher consumption of vegetables, fruits and dietary fibre^(21)^. Lower snack ED was positively associated with diet quality but not with the BMI Z-score^(16)^. Even though snack ED is related to the consumption of nutritious snacks, it is not a direct measure of snack nutritional quality, but rather a proxy for the nutritional quality of snacks. Snack nutritional quality remains an understudied characteristic of snacks owing to the lack of established tools to specifically examine the nutritional quality of snacks^22^.

The British Food Standards Agency nutrient profiling system was created in 2004–2005 to provide a way to distinguish between foods based on their nutritional value for the purpose of regulating food advertising to children on television. The nutritional profiling system examines foods to be advertised on television based on their content of energy, saturated fatty acids, total sugar, Na, fruits/vegetables/nuts, dietary fibre and protein^(23)^. The study by Murakami^(22)^ revealed no significant association between the nutritional quality of snacks, as evaluated by the British Food Standards Agency nutrient profiling system, diet quality (as measured by the Mediterranean dietary index) and BMI Z-score. However, to date, no studies have examined the relationship between snack nutritional quality, diet quality and adiposity in adolescent populations outside UK.

The Nutrient Profiling Scoring Criterion (NPSC) is a nutrient profiling system originally developed in Australia and New Zealand to determine whether a food can make a health claim based on its nutrient profile^(24)^. The NPSC can be used as an alternative snack nutritional quality assessment system in Australia. Examining the association between the nutritional quality of snacks (as evaluated by NPSC), diet quality and adiposity, as well as the capacity of NPSC to distinguish between unhealthy and healthy snacks, is crucial to determine its use in evaluating snack nutritional quality. Hence, this study aimed to examine the association between nutritional quality of snacks assessed using NPSC with overall diet quality and adiposity among Australian adolescents. In addition, the distribution of discretionary foods (i.e. energy-dense and nutrient-poor foods and beverages) and intake from the five food groups at different levels of snack nutritional quality was examined.

## Methods

### Sample and study design

This cross-sectional study utilised data from the 2011–2012 National Nutrition and Physical Activity Survey (NNPAS). The sampling method and survey design have been described in detail elsewhere^(25)^. Briefly, the NNPAS was conducted nationwide by the Australian Bureau of Statistics from May 2011 to June 2012. The survey used a multistage stratified probability sampling design to recruit participants from private households in Australia. For each participating household, one adult (aged 18 years or older and a regular resident) and, if applicable, one child aged 2–17 years were randomly selected. Socio-demographic, dietary and anthropometric data were gathered from the participants through face-to-face interviews^(25)^.

The final sample comprised 9519 households, representing a household response rate of 77 %. In total, 12 153 participants were selected from these households. Among them, 1101 were adolescents between the ages of 12 and 18 years.

The Census and Statistics Act 1905 provides ethics approval for the Australian Bureau of Statistics to carry out household interviews and anthropometric measurements for surveys. In this study, non-identifiable data were used for secondary analysis. The Deakin University Human Research Ethics Committee approved an exemption from ethics review for this study under application number 2023-193.

### Analytic sample

**Fig. 1.**
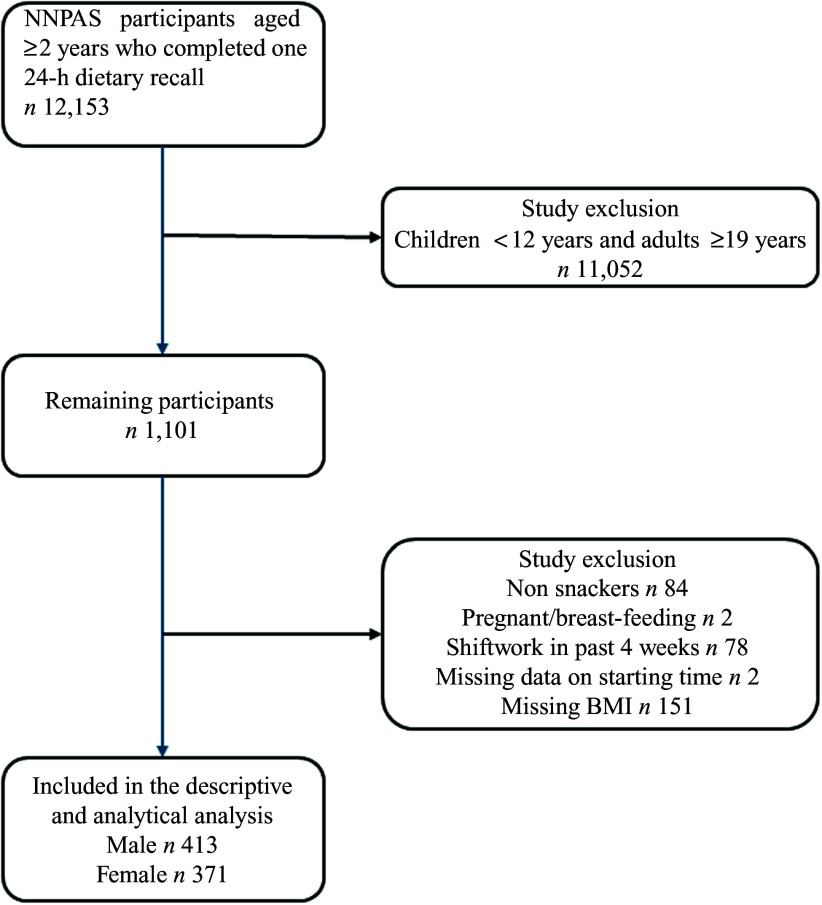
Flow diagram of participants included in the analysis.

The present study included adolescents aged 12–18 years who completed their first 24-h dietary recall. Pregnant or breast-feeding individuals (*n* 2) and those adolescents aged 14–18 years who had engaged in shift work (*n* 78) in the past 4 weeks (e.g. night or rotating shifts affecting eating patterns) were excluded. A total of 1021 adolescents were initially eligible for inclusion in the study. Adolescents were further excluded if they did not report their time of eating (*n* 2) or if they did not report consuming snacks in their 24-h dietary recall (*n* 84). In addition, adolescents with missing BMI have been excluded (*n* 151). The association between snack nutritional quality and overall diet quality, BMI Z-score and waist circumference included 784 adolescents (413 boys and 371 girls). A schematic representation of the final sample determination is provided in Fig. 1. The differences in the characteristics of adolescents included in this study and those who were excluded due to missing BMI data were examined, and the results can be found in online Supplementary File 2. The results showed no significant differences in age, sex, area-level disadvantage, meeting physical activity guidelines, snack characteristics and Dietary Guideline Index for Children and Adolescents (DGI-CA) scores between those who were excluded from further analysis and those who were included.

### Dietary assessment

The NNPAS collected dietary data through 24-h dietary recalls using the validated U.S. Department of Agriculture, 5-pass multiple recall method^(26,27)^. Participants indicated the type of each eating occasion (EO, from a list of response options), along with their corresponding initiation time for each food and beverage consumed^(26–28)^. The two dietary recalls were collected on non-consecutive days across all seasons, the first recall was conducted through face-to-face interviews and the second recall was conducted in 60 % of the total sample via telephone after at least 8 days. The Australian Supplement and Nutrient Database 2011–2013 was used to calculate energy and nutrient intake^(29)^. The Australian Supplement and Nutrient Database 2011–2013 was used to calculate energy intake (kJ) and nutrient intake (g) from all foods and drinks reported during the recalls^(30)^. The dietary information collected from the first day of recall was used to maintain national representativeness and maximise adolescent participation.

### Snack definition

The EO identified by participants as snacks, morning/afternoon tea and beverage breaks were classified as snacks. EO self-reported as breakfast, brunch, lunch, dinner and supper were considered as meals. EO reported as ‘extended consumption’ or ‘other/don’t know’ were categorised as meals and snacks if they occurred concurrently or within 15 min of an EO reported as a meal or a snack, respectively. Similarly, following this, all EO of the same category, occurring within 15 min of each other were combined and treated as a single EO (e.g. two snack EOs occurring within 15 min were combined into one snack)^(31)^. After combining EO, any EO with less than 210 kJ energy were excluded from the analysis. This participant-identified approach to defining EO coupled with the additional criteria of a 15-min time interval, and a 210-kJ minimum has been previously shown to predict the most variation in total energy intake among Australian children and adolescents^(31)^.

### Snack characteristics

Several snack characteristics were examined, including snack frequency, snack ED and snack nutritional quality. Snack frequency includes the number of snacks consumed by each participant. Two different methods were used to calculate the ED of snacks and meals. The first method included all foods and beverages, and the other method excluded liquids consumed as beverages. The calculation of ED excluding beverages followed the method proposed by Vernarelli, Mitchell^(32)^. In summary, beverages containing energy and those without energy were omitted, but tap water and milk (including non-dairy milk substitutes) were treated differently because these beverages are often consumed as ingredients or as additional components in other food items (e.g. milk added to ready-to-eat cereals or tap water added to cooked cereals). Milk and water consumed as ingredients were included as foods in the ED calculations. The ED (including all food and beverages, excluding beverages) was calculated by first determining the daily total energy and daily weight derived from snacks or meals, including all food and beverages, and then by excluding beverages. Next, the total energy from the snack or meal was divided by the corresponding total weight to obtain the ED.

Snack and meal nutritional quality were assessed using the NPSC^(24)^. A detailed description of the procedure for calculating the NPSC score has been published previously^(24)^. Briefly, the NPSC score was calculated for each food and beverage consumed based on the nutritional content per 100 g. Foods and beverages were categorised into three categories. The first category included beverages. The second category includes foods and beverages that are not included in the first or third categories. The third category includes cheeses and processed cheeses with > 320 mg calcium/100 g, edible oil, edible oil spreads, margarine and butter. The NPSC has two major components called ‘baseline points’ and ‘modifying points.’ Baseline points were the sum of the scores allocated for energy, saturated fat, Na and sugar content. The scores for each component ranged from 0 to 30 or 50 depending on the category. A higher score indicates a higher content of baseline components. Modifying points are the sum of the scores allocated for fruit, vegetables and nuts, protein and fibre. The scores for each component range from 0 to 8. A higher score indicates a higher content of modifying point components. The final score was obtained by subtracting the modifying points from the baseline points. The final score can range from -13 (most healthy) to 61 (least healthy)^(24)^. This detailed description of the steps for calculating the NPSC score can be found in online Supplementary File 1.

To aggregate the NPSC scores of foods and beverages for snacks and meals, the NPSC score of each food or beverage was multiplied by its corresponding energy, and these values were then summed per snack or meal. Subsequently, the sum was divided by the total energy intake (EI) of the snacks or meals. This procedure was performed according to the procedure described by Murakami^(22)^.

Food groups consumed at snack were further classified into two major groups: those belonging to five food groups and those belonging to discretionary foods. The five food groups consisted of: (1) vegetables and legumes/beans; (2) fruit; (3) grain (cereal) foods, primarily wholegrain and/or high cereal fibre varieties; (4) lean meats, poultry, fish, eggs, tofu, nuts and seeds and legumes/beans and (5) milk, yogurt, cheese and/or alternatives, mainly reduced-fat options. In contrast, discretionary foods include choices that are high in energy, saturated fat, added sugars and/or salt or those containing alcohol^(30)^.

### Diet quality assessment

The DGI-CA was used to assess the dietary quality of adolescents using first-day recall^(33)^. The specifics regarding its components, along with the development and evaluation of the DGI-CA, have been thoroughly explained elsewhere^(33)^. The DGI-CA measures how well children and adolescents adhere to the 2013 Australian Dietary Guidelines^(30,33)^. It consists of nine components, seven of which evaluate adequate intake: (1) consuming a variety of nutritious foods; (2) including vegetables, legumes and beans; (3) consuming mostly whole grains and/or high-fibre grains; (4) consuming fruits; (5) including lean meats, poultry, fish, eggs, tofu, nuts/seeds and legumes/beans; (6) consuming dairy products and/or alternatives, mostly low-fat options and (7) drinking an ample amount of water. The remaining two components assessed limited intake: limiting the intake of saturated fats, alcohol, added salt and sugars and replacing saturated fats with unsaturated fats. The scores from these nine components were summed to obtain a total score, with a maximum of 100 points. Higher scores indicate better diet quality.

### Evaluation of energy intake reporting

To assess the potential energy misreporting, we employed the method proposed by Huang, Roberts^(34)^. This method involves calculating the ratio of EI to estimated energy requirements (EER). The EER is determined using established Dietary Reference Intake equations, which consider factors such as sex, age, body weight, height and level of physical activity^(35)^. A ‘low active’ level of physical activity (≥ 1·4 to < 1·6) was assumed for all adolescents due to a lack of information on adolescents’ total daily physical activity level (i.e. lack of data on non-leisure time physical activity) in the NNPAS. A synthesis of state-level and national data showed that a majority of adolescents in Australia did not meet the daily minimum of 60 min of moderate- to vigorous-intensity physical activity on most or all days of the week^(36)^. Similarly, 93 % of adolescents in this study did not meet the recommended physical activity guidelines. Thus, assigning low-active values of physical activity may be a reasonable assumption with minimal impact on EER estimates. In the regression models, the EI: EER ratio was used as a covariate to adjust for potential energy misreporting.

### Anthropometry

Anthropometric measurements of the NNPAS were performed voluntarily. Digital scales with a maximum weight limit of 150 kg were used to measure weight, whereas height was measured using a stadiometer up to 210 cm. The participants were encouraged but were not required to remove their shoes and heavy clothing before being measured. The impact of clothing on measurements was not recorded. If a participant reported a weight exceeding the maximum measurement capacity of the scale (150 kg), their weight was not recorded. Weight was recorded in kilograms with one decimal place, and height and waist measurements were recorded in centimetres with one decimal place^(37)^.

Waist circumference was measured following the guidelines provided by the WHO^(38)^, while ensuring minimal discomfort for the participants. According to the WHO, waist circumference should be measured at the midpoint between the lower margin of the least palpable rib and the top of the iliac crest. To measure this, NNPAS interviewers held the end of the tape at an appropriate point and asked the respondent to turn around until the tape met or they asked the respondent to hold the end of the tape while they walked around them until the tape met. A metal measuring tape with a maximum length of 200 cm was used to prevent stretching of the tape and to ensure accurate measurements.

To verify the accuracy of the height and waist measurements, a random 10 % of the respondents were selected for duplicate measurements. If the second measurement of height or waist differed by more than one centimetre, a third reading was recorded. However, weight measurements were collected only once. The BMI z-score was computed using the WHO growth reference population^(39)^.

Waist:height ratio (WHtR) was computed as the ratio of waist circumference to height.

### Covariates

This study explored several potential covariates, such as age, area-level disadvantage, adolescents’ compliance with physical activity guideline, meal frequency and meal nutritional quality, owing to their demonstrated influence on the association between adolescent snack characteristics, overall diet quality and adiposity^(22)^. Meal frequency included the number of meals consumed by each participant for the recall day, which was determined using the approach described earlier. Meal nutritional quality is measured by the NPSC score following the approach described above. Participants reported their age in years. Participants’ household postal codes were used to determine the Socio-Economic Index for Areas, which measures the level of disadvantage within specific regions. The Socio-Economic Index for Areas scores range from one (indicating the highest level of disadvantage) to five (representing the most advantaged)^(25,31)^. Adolescents aged 12–17 years who adhered to the guideline of engaging in a minimum of 60 min of moderate to vigorous physical activity each day and limited their screen time to a maximum of 2 h for entertainment or non-educational purposes within the past week were considered to have fulfilled the physical activity recommendation. For 18-year-old adolescents, those who participated in physical activity for at least 150 min across five or more weekly sessions were categorised as meeting the physical activity recommendation^(37)^.

### Data analysis

All statistical analyses were performed using the appropriate NNPAS person and replicate weights to account for the complex survey design and the probability of selection^(25)^. All statistical analyses were conducted using Stata 17. Descriptive statistical analyses were performed using frequencies and proportions for categorical variables. Continuous variables were described using means and 95 % CI. The mean difference was tested using an F-test, while the differences in proportion were tested using the adjusted Wald test and the *χ* ^2^ test. Snacks NPSC score was stratified into tertiles and then the mean intake (serves) of foods from the five food groups and discretionary foods at snacks were examined across tertiles of snack nutritional quality. Foods identified as discretionary foods were excluded from the calculation of food intake (serves) from the five food groups consumed at snacks. Adolescents in the 1st tertile were considered to have high snack nutritional quality, whereas those in the second tertile were considered to have medium snack nutritional quality, and those in the third tertile were considered to have low snack nutritional quality. All statistical analyses were stratified by sex due to the previously reported variation in snack characteristics by sex, particularly snack frequency and food groups consumed at snack^(12,14,40–42)^.

Multiple linear regression was used to investigate the association between snack nutritional quality (as measured by the NPSC score) and overall diet quality (DGI-CA score; continuous). Model covariates were selected based on their associations with the exposure and outcome variables, as reported in the literature^(22,43)^. Multiple multivariate models were used to observe any changes in strength and direction of association with the addition of covariates to the model (e.g. to observe the impact of energy misreporting (EI:EER) on the measure of association). Model assumptions were checked and found to be met, including an assessment of multicollinearity^(22,43)^. The first model was unadjusted, followed by a second model that was adjusted for age, sex, area-level disadvantage (Socio-Economic Index for Areas) and whether physical activity guidelines were met. The third model was adjusted for meal nutritional quality (as measured by the NPSC score), snack frequency and meal frequency. The fourth model was adjusted for EI: EER to account for energy misreporting.

The association between snack nutritional quality, BMI z-score, waist circumference and WHtR was investigated using linear regression, adjusting for the same covariates and procedures as stated above for the DGI-CA score. Waist circumference was log-transformed to meet the model assumptions. Statistical significance was set at *P* < 0·05.

## Results

Table 1 presents the characteristics of the adolescents. There were no differences in average age, BMI Z-score, NPSC score for snacks, NPSC score for meals or DGI-CA score between boys and girls. However, boys had a significantly higher waist circumference than girls.

**Table 1.** Characteristics of adolescents between 12 and 18 years in the National Nutrition and Physical Activity Survey 2011–2012 (Weighted percentages, weighted means and 95 % confidence intervals)

	Boys (*n* 413)		Girls (*n* 371)		*P* †
Characteristics	Mean	95 % CI	Mean	95 % CI	
Age (Years)	14·7	14·5, 15	14·6	14·3, 14·9	0·61
Area-level disadvantage (SEIFA)* quintiles. (%)					
Lowest 20 %	17·6	12·9, 23·5	12·6	7·8, 19·8	0·23
Second quintile	19·4	14·2, 26·0	18·3	13·3, 24·7	
Third quintile	19·6	13·9, 26·8	27·3	19·5, 36·8	
Fourth quintile	18·8	14·0, 24·8	19·0	14·0, 25·3	
Highest 20 %	24·7	18·4, 32·2	22·7	17·3, 29·2	
BMI Z-Score	0·6	0·4, 0·7	0·5	0·3, 0·6	0·48
Waist circumference	78·6	77, 80·3	73·9	72·6, 75·2	< 0·001
WHtR	0·46	0·46, 0·47	0·46	0·45, 0·46	0·17
Meets physical activity guideline (%)	9·9	6·5 %, 14·9	7·7	17·3, 29·2	0·15
Snack frequency	2·5	2·3, 2·6	2·3	2·1, 2·4	0·08
ED of meals including beverages	6·2	6, 6·5	6·3	6, 6·7	0·67
ED of meals without beverages	6·4	6·1, 6·6	6·5	6·1, 6·8	0·71
ED of snacks including beverages	6·9	6·3, 7·6	7·7	7·1, 8·3	0·08
ED of snacks without beverages	9·2	8·4, 10	9·1	8·4, 9·7	0·73
NPSC of meals	4·5	3·9, 5·1	4·6	3·9, 5·3	0·83
NPSC of snacks	10·2	9, 11·4	10·3	9·2, 11·5	0·85
DGI-CA score	45·1	43·2, 47	44·0	42·1, 45·9	0·48

ED, energy density; WHtR, waist:height ratio..

NPSC, Nutrient Profiling Scoring Criterion, score can range from −13 (most healthy) to 61 (least healthy)^(24)^.

DGI-CA, Dietary Guideline Index – Children and Adolescents^(33)^.

Results are presented as weighted percentages (%) or weighted means (95 % CI).

* Australian Bureau of Statistics Socio-Economic Indexes for Areas. SEIFA quintiles range from one (most disadvantaged) to five (most advantaged).

† *P* value for differences between boys and girls based on F test (continuous variable), adjusted Wald test (categorical variables) and *χ* ^2^ test (area level disadvantage).

Among both boys and girls, the highest intake of fruits, vegetables and legumes/beans at snacks was observed among adolescents with the highest snack nutritional quality score, and the intake of these foods increased with improvement in snack nutritional quality. Conversely, the lowest intake of discretionary foods at snacks was observed among both boys and girls with high snack nutritional quality, and the intake of these foods decreased with improvement in snack nutritional quality. There was no notable increase or decrease in the intake of grain (cereal) foods, mostly wholegrain and/or high cereal fibre varieties and milk, yoghurt cheese, and/or alternatives and mostly reduced fat at snacks across snack nutritional quality among adolescents (Table 2).

**Table 2.** Distribution of food groups across snack nutritional quality of adolescent boys between 12 and 18 years in the National Nutrition and Physical Activity Survey 2011–2012 (Mean values and 95 % confidence intervals)

	Snack nutritional quality (NPSC score for snacks)†,‡					
	Low		Medium		High	
Food group (Serves)* ^,^ §	Mean	95 % CI	Mean	95 % CI	Mean	95 % CI
Boys						
Fruit	0·00	0·00, 0·00	0·00	0·00, 0·01	0·39	0·31, 0·46
Fruits (excluding fruit juice)	0·00	0·00, 0·00	0·00	0·00, 0·01	0·29	0·22, 0·36
Fruit juice	0·00	0·00, 0·00	0·00	0·00, 0·00	0·10	0·04, 0·16
Vegetables and legumes/beans	0·00	0·00, 0·01	0·01	0·00, 0·01	0·02	0·00, 0·04
Grain (cereal) foods, mostly wholegrain and/or high cereal fibre varieties	0·07	–0·01, 0·15	0·17	0·10, 0·24	0·20	0·14, 0·26
Wholegrain or high fibre cereal/grains	0·00	0·00, 0·00	0·03	0·00, 0·06	0·05	0·02, 0·08
Refined or lower fibre cereals/grains	0·07	–0·01, 0·15	0·14	0·08, 0·19	0·15	0·09, 0·20
Milk, yoghurt cheese and/or alternatives, mostly reduced fat	0·07	0·03, 0·10	0·17	0·11, 0·22	0·08	0·05, 0·11
Lean meats and poultry, fish, eggs, tofu, nuts and seeds and legumes/beans	0·01	0·00, 0·02	0·04	0·01, 0·06	0·03	0·02, 0·05
Discretionary foods	1·18	1·02, 1·34	0·41	0·32, 0·49	0·02	0·00, 0·04
Girls						
Fruit	0·00	0·00, 0·00	0·01	0·00, 0·02	0·30	0·24, 0·37
Fruits (excluding fruit juice)	0·00	0·00, 0·00	0·01	0·00, 0·02	0·24	0·19, 0·30
Fruit juice	0·00	0·00, 0·00	0·00	0·00, 0·00	0·06	0·02, 0·09
Vegetables and legumes/beans	0·00	0·00, 0·00	0·00	0·00, 0·00	0·04	0·02, 0·07
Grain (cereal) foods, mostly wholegrain and/or high cereal fibre varieties	0·05	0·00, 0·11	0·11	0·06, 0·16	0·12	0·07, 0·16
Wholegrain or high fibre cereal/grains	0·00	0·00, 0·00	0·01	0·00, 0·02	0·03	0·01, 0·05
Refined or lower fibre cereals/grains	0·05	0·00, 0·11	0·10	0·05, 0·15	0·08	0·04, 0·13
Milk, yoghurt cheese and/or alternatives, mostly reduced fat	0·04	0·01, 0·06	0·13	0·09, 0·17	0·07	0·03, 0·11
Lean meats and poultry, fish, eggs, tofu, nuts and seeds and legumes/beans	0·01	–0·01, 0·03	0·02	0·01, 0·04	0·02	0·00, 0·03
Discretionary foods	0·91	0·75, 1·07	0·32	0·25, 0·38	0·01	0·00, 0·01

* The mean intake of food groups was calculated after excluding foods that include discretionary foods.

† Snack nutritional quality (NPSC score) among boys: Highest snack nutritional quality = −12–0; medium snack nutritional quality = 0·02–2·05; low snack nutritional quality = 2·06–45.

‡ Snack nutritional quality (NPSC score for snacks) among girls: High snack nutritional quality = −11–0; medium snack nutritional quality = 0·01–1·9; low snack nutritional quality = 2–47.

§ A serving of fruit provides 350 kJ, a serving of vegetables and legumes/beans ranges from 100 to 350 kJ, a serving of grain (cereal) foods, mostly wholegrain and/or high cereal fibre varieties, offers 500 kJ, and a serving of milk, yogurt, cheese and/or alternatives, mostly reduced fat, along with lean meats, poultry, fish, eggs, tofu, nuts, seeds and legumes/beans, will provide approximately 500–600 kJ, while a serving of discretionary foods equals 600 kJ.

Higher nutritional quality of snacks, as assessed by the NSPC, was associated with higher diet quality measured by the DGI-CA in both boys and girls. A one-point increase in the NPSC score of snacks (indicating a reduction in snack nutritional quality) was associated with a 0·48-point decrease in the DGI-CA score (*P* < 0·001). Moreover, in the adjusted model, boys had lower *β*-estimates compared with girls (Table 3).

**Table 3.** Association of snack nutritional quality with DGI-CA among adolescents between 12–18 years from the National Nutrition and Physical Activity Survey 2011–2012 (*β* and 95 % confidence intervals)

	1st model†		2nd model‡		3rd model§		4th model||	
	*β*	95 % CI	*β*	95 % CI	*β*	95 % CI	*β*	95 % CI
Boys (*n* 413)								
NPSC of snacks	–0·55***	–0·75, −0·35	–0·55***	–0·76, −0·35	–0·53***	–0·68, −0·38	–0·53***	–0·70, −0·36
Girls (*n* 371)								
NPSC of snacks	–0·37***	–0·52, −0·21	–0·37***	–0·52, −0·22	–0·36***	–0·52, −0·21	–0·42***	–0·58, −0·26

NPSC, Nutrient Profiling Scoring Criterion. The score ranges from −13 (most healthy) to 61 (least healthy)^(24)^.

*P* values: *P > 0·05, **P < 0·05, **P < 0·01, ***P < 0·001.

† Unadjusted model.

‡ Adjusted for age, area-level disadvantage (SEIFA), meeting physical activity guidelines.

§ Adjusted for variables in model 2, meal nutritional quality (as measured by NPSC score), snack frequency and meal frequency.

|| Adjusted for variables included in model 2, model 3 and EI: EER.

Table 4 shows the association between snack nutritional quality (NPSC score) with BMI Z-score waist circumference and WHtR, respectively. After adjusting for age, sex, area-level disadvantage, meal nutritional quality (as measured by the NPSC score), meeting the physical activity guidelines and EI: EER, no significant association was observed between snack nutritional quality (NPSC score) and either BMI Z-score waist circumference among boys or girls (all *P* > 0·05). Moreover, across all models assessing the association between snack nutritional quality and both BMI Z-score waist circumference and WHtR, no significant associations were found.

**Table 4.** Association of snack nutritional quality with BMI Z-score, waist circumference and WHtR among adolescents between 12 and 18 years from the National Nutrition and Physical Activity Survey 2011–2012 (*β* and 95 % confidence intervals)

	1st model†		2nd model‡		3rd model§		4th model||	
	*β*	95 % CI	*β*	95 % CI	*β*	95 % CI	*β*	95 % CI
BMI Z-score								
Boys (*n* 413)								
NPSC of snacks	–0·001*	–0·016, 0·019	0·001*	–0·017, 0·019	–0·016*	–0·016, 0·019	–0·007*	–0·009, 0·02
Girls (*n* 371)								
NPSC of snacks	–0·011*	–0·026, 0·005	–0·010*	–0·026, 0·006	–0·010*	–0·025, 0·005	–0·006*	–0·021, 0·08
Waist circumference								
Boys (*n* 406)								
NPSC of snacks	–0·002*	–0·004, 0·000	0·000*	–0·002, 0·001	0·000*	–0·002, 0·001	0·000*	–0·001, 0·002
Girls (*n* 367)								
NPSC of snacks	–0·001*	–0·003, 0·000	–0·001*	–0·003, 0·001	–0·000*	–0·002, 0·000	–0·000*	–0·002, 0·001
WHtR								
Boys (*n* 406)								
NPSC of snacks	0·000*	–0·001, 0·000	0·000*	–0·001, 0·001	0·000*	–0·001, 0·001	0·000*	–0·001, 0·001
Girls (*n* 367)								
NPSC of snacks	0·000*	–0·001, 0·000	0·001*	–0·001, 0·000	0·000*	–0·001, 0·001	0·000*	–0·001, 0·001

WHtR, waist:height ratio.

Waist circumference was log-transformed to improve normality. The format for interpretation of the b-coefficient estimates is therefore 100 × (coefficient), corresponding to the percentage change for a 1-unit increase in the independent variable (while holding all other variables constant).

NPSC: Nutrient Profiling Scoring Criterion. The score ranges from −13 (most healthy) to 61 (least healthy)^(24)^.

*P* values: *P > 0·05, **P < 0·05, **P < 0·01, ***P < 0·001.

† Unadjusted model.

‡ Adjusted for age, area-level disadvantage (SEIFA), meeting physical activity guideline.

§ Adjusted for variables in model 2, meal nutritional quality (as measured by NPSC score), snack frequency and meal frequency.

|| Adjusted for variables included in model 2, model 3 and EI: EER.

## Discussion

To our knowledge, this is the first study to apply the NPSC score to assess snack nutritional quality in adolescents and examine its association with diet quality, and adiposity, as well as the capacity of NPSC to distinguish between unhealthy (e.g., high-energy, low-nutrient ‘discretionary’ foods and drinks) and healthy (e.g. foods from the five food groups) snacks. We found that snack nutritional quality, as assessed using NPSC score, was associated with better diet quality and can be used to identify snacks with a better nutritional profile among adolescents. However, no association was observed between snack nutritional quality and adiposity as measured using the BMI Z-score, waist circumference and WHtR. Furthermore, better snack nutritional quality was accompanied by lower consumption of discretionary foods at snack and higher consumption of fruits at snack. These findings indicate that snack nutrition quality measured by NPSC has the potential to identify snacks with better nutritional quality that could enhance adolescent overall dietary quality.

The distribution of foods from the five food groups and discretionary foods varied across levels of snack nutritional quality. Discretionary food intake at snacks decreased with an improvement in the nutritional quality of snacks. This might be because energy, saturated fat and Na content have been included as NPSC components which are also the hallmarks of discretionary foods^(30)^. Fruit intake increased with an increase in snack nutrition quality, which might be due to the low energy content and high fibre content of fruits which are positively scored in the NPSC score^(24)^. However, there were no observable differences in milk intake across levels of snack nutritional quality. This might be because full-fat milk is commonly consumed as a snack, and the NPSC score penalises fat intake, irrespective of the food source. The Australian Dietary Guidelines recommend the consumption of reduced-fat dairy products for children (two year and above) and adolescents due to the need to balance energy requirements^(30)^. However, according to a position statement from the National Heart Foundation of Australia, unflavoured milk is among the healthy snack choices from dairy products as long as the primary source of fat in the diet is from foods such as fish, olives, seeds, nuts and oils made from them^(44)^. Considering this, future snack nutritional quality assessment tools may need to consider alternative approaches to scoring fat content, particularly considering the most recent evidence on dairy among adolescents^(44)^. Contrary to expectations, a high intake of refined grain was observed among those with better snack nutritional quality. This might be because the NPSC score did not include components related to the intake of refined grains. To address the limitations of snack nutritional quality evaluated via nutrient profiling systems, which do not account for the source of fat or the difference between refined and whole-grain products, a food-based profiling system could be employed to assess snack nutritional quality.

Higher nutritional quality of snacks, as assessed by the NSPC, was associated with higher diet quality (DGI-CA) in both boys and girls. While a stronger association with diet quality was initially observed among girls, compared with boys. However, this effect size observed in girls became smaller when adjusting for energy misreporting. This lower effect size was not observed in boys. The sex differences in the strength of association may be attributed to the varying impact of energy misreporting between boys and girls^(45,46)^. A study by Murakami^(22)^ examining the relationship between Mediterranean diet scores and the nutritional quality of meals and snacks, assessed using the Food Standards Agency nutrient profiling system, found a similar association among British adolescents. However, the association with snack nutritional quality did not reach statistical significance. This observed association may be attributed to shared components between DGI-CA and NPSC^(24,33)^, including the intake of vegetables, saturated fats and indirectly, fibre intake, which is assessed in the NPSC. This fibre intake may be related to the components of DGI-CA that give favourable scores to foods with a high fibre content, including the consumption of nutritious foods such as fruits, vegetables and whole grains^(33)^. This association between snack nutritional quality and diet quality suggests the potential use of snack nutritional quality as an indicator to identify snacks that contribute favourably to overall diet quality. Furthermore, it is important to note that snack characteristics and the ideal amount of food groups needed from snacks may vary depending on age and sex^(47)^. Therefore, snack nutritional quality indices should be tailored to specific age and sex groups. However, nutrient profiling scores currently available for use as snack nutritional quality indices are neither sex nor age specific. Currently, existing nutritional profiling systems with the potential to assess snack nutritional quality have been associated with diet quality and discriminate between discretionary and nutritious foods consumed at snacks^(22)^. However, these nutritional profiling systems have not been developed considering optimum snack characteristics (snack frequency, snack ED and food groups consumed at snack) that are linked with improved overall diet quality.

The nutritional quality of snacks was found to have no significant association with adiposity, as measured by BMI Z-score, waist circumference and WHtR. A similar study conducted with British adolescents showed comparable results when examining the relationship between snack nutritional quality, as assessed using the British Food Standards Agency nutrient profiling system, and adiposity, measured using BMI Z-scores and waist circumference^(22)^. On the other hand, a study by Murakami^(43)^ conducted among British adults showed that BMI and waist circumference in women increase with lower nutritional quality of snacks that are identified based on EI contribution. A related study conducted in French adults showed that the British Food Standards Agency nutrient profiling score, NPSC, Health Star Rating nutrient profiling score and French High Council of Public Health NPS nutrient profiling score applied for the whole diet were associated with increased BMI over time^(48)^. These mixed findings, with the absence of an association observed in studies involving nutrient profiling scores and adolescent adiposity, along with the significant association between nutrient profiling scores in adults, may be attributed to the fact that these nutrient profile scores are not age- and sex-specific, even though energy and nutrient intake associated with adiposity are dependent on age and sex^(49–51)^. In addition, this discrepancy between findings from studies conducted in adolescents and adults might also be attributed to differences in metabolic rate and energy requirements. Adolescence is characterised by higher metabolic rate due to growth spurts and development^(52,53)^. This elevated metabolic rate and energy demand may allow adolescents to utilise energy and nutrients from snacks more efficiently, possibly mitigating some of the adverse effects of snacks with lower nutritional quality on BMI Z-score and waist circumference. In contrast, adults typically have lower metabolic rates and may store excess energy from snacks with lower nutritional quality as fat, leading to increased BMI Z-score and waist circumference^(52,53)^. Furthermore, despite the impact of the level of physical activity on adolescent adiposity^(54,55)^, this study only adjusted for whether adolescents met the recommended physical activity level, but this variable may not have captured the full variation in adolescents’ total daily physical activity levels.

This study has several notable strengths. First, it employed a comprehensive analysis of a relatively underutilised nutrient profiling score to assess the nutritional quality of snacks. The data gathered on dietary habits came from a nationally representative sample of Australian adolescents and were collected throughout the year. This may allow for the consideration of potential variations in snacking characteristics and diet quality, both on school terms and school holiday, as well as across different seasons among adolescents. The models were adjusted for potential energy misreporting. However, the calculations for energy intake to estimated energy requirement (EI: EER) assumed that all adolescents fell into the ‘low-active’ physical activity group. It is important to recognise that a considerable number of adolescents do not reach the recommended minimum of 60 min of moderate to vigorous physical activity on most days, assigning low-intensity physical activity values may not have a significant impact^(36)^.

The findings of this study should be interpreted with consideration of the following limitations. There is a lack of tools developed with the purpose of assessing the nutritional quality of snacks. Although existing nutrient profiling scores can potentially be used to assess snack nutritional quality, they have not been developed considering the characteristics of snacks linked to optimal overall diet quality or excess adiposity. The use of a single day’s dietary data from a 24-h dietary recall may not accurately capture the day-to-day variation in snack characteristics. However, a study conducted during the NNPAS 2011–2012 found no significant differences in snack frequency or energy intake contribution from snacks between adolescents who completed one or both days of recall^(31)^. Moreover, weekends are underrepresented in the 24 h dietary recall, which may lead to a failure to capture the variation in snack characteristics across weekends. The survey was carried out between 2011 and 2012 that may not represent current Australian adolescents snacking; however, it is the latest available data. With the next survey data expected to be released in 2025, the findings of this study can be used to track changes in adolescent snack characters over time^(11,42)^. The findings could be extended to other high-income countries like the USA and UK that have reported similar patterns of adolescent snack characteristics and lower diet quality among adolescents^(11,15,22,41)^. In addition, more recent studies from the USA have also showed that snacking is still prevalent among adolescents and that snacks comprise mostly unhealthy foods^(11,41)^.

### Conclusion

In summary, increase in snack quality was associated with better overall diet quality in both adolescent boys and girls. Snacks with higher nutritional quality were associated with lower intake of discretionary foods and higher consumption of fruits. However, there is a lack of association between snack nutritional quality and measures of adiposity. Future studies should aim to develop a food-based profiling system that examines snack nutritional quality considering the characteristics of snacks such as snack frequency, snack ED and food groups consumed as snack that are related to optimal diet quality and adiposity with sex and age variations in mind.

## Supporting information

Sisay et al. supplementary material 1 Sisay et al. supplementary material

Sisay et al. supplementary material 2 Sisay et al. supplementary material

## Supplementary material

For supplementary material/s referred to in this article, please visit https://doi.org/10.1017/S0007114524001727
