# Supplementary material for: Associations between the nutritional quality of snacks, overall diet quality and adiposity: findings from a nationally representative study of Australian adolescents
Source: Br J Nutr. 2024 Sep 18;132(4):522–31. doi: 10.1017/S0007114524001727 (PMC11499082; doi:10.1017/S0007114524001727)
Supplement: Sisay et al. supplementary material 1 — Sisay et al. supplementary material [file S0007114524001727sup001.docx]

**NPSC**^(2)^

The Nutrient Profiling Scoring Criterion (NPSC) evaluates the nutritional content of foods and beverages based on 100g (or 100 mL) for unfavourable elements such as energy, saturated fats, sugars, and sodium, which contribute to a total of baseline points. Conversely, favourable elements such as proteins, fibres, fruits, vegetables, and nuts contribute to a total of modifying points.

**Food categories:**

The NPSC considers three different food categories for the score calculation:

- Category 1: beverages
- Category 2: all foods not included in category 1 or category 3
- Category 3: cheeses and processed cheeses with >320mg calcium/100g, edible oil, edible oil spreads, margarine and butter

**Step 1: Calculation of baseline points**

Baseline points = [points of energy] + [points of sat fat] + [points of total sugar] + [points of sodium]

- Categories 1 and 2: Scored to maximum of 10 points for the baseline components.
- Category 3: Foods in this category can be scored beyond 10 points. The maximum points for the baseline components are 11 points for energy, 30 points for saturated fat, and 30 points for sodium.

| ***Points*** | **Energy (kJ)** | **Saturated Fat (g)** | **Total Sugars (g)** | **Sodium (mg)** |
| --- | --- | --- | --- | --- |
| 0 | ≤ 335 | ≤ 1 | ≤ 5 | ≤ 90 |
| 1 | > 335 | > 1 | > 5 | > 90 |
| 2 | > 670 | > 2 | > 9 | > 180 |
| 3 | > 1005 | > 3 | > 13.5 | > 270 |
| 4 | > 1340 | > 4 | > 18 | > 360 |
| 5 | > 1675 | > 5 | > 22.5 | > 450 |
| 6 | > 2010 | > 6 | > 27 | > 540 |
| 7 | > 2345 | > 7 | > 31 | > 630 |
| 8 | > 2680 | > 8 | > 36 | > 720 |
| 9 | > 3015 | > 9 | > 40 | > 810 |
| 10 | > 3350 | > 10 | > 45 | > 900 |
| 11 | > 3685 | > 11 |  | > 990 |
| 12 |  | > 12 |  | > 1080 |
| 13 |  | > 13 |  | > 1170 |
| 14 |  | > 14 |  | > 1260 |
| 15 |  | > 15 |  | > 1350 |
| 16 |  | > 16 |  | > 1440 |
| 17 |  | > 17 |  | > 1530 |
| 18 |  | > 18 |  | > 1620 |
| 19 |  | > 19 |  | > 1710 |
| 20 |  | > 20 |  | > 1800 |
| 21 |  | > 21 |  | > 1890 |
| 22 |  | > 22 |  | > 1980 |
| 23 |  | > 23 |  | > 2070 |
| 24 |  | > 24 |  | > 2160 |
| 25 |  | > 25 |  | > 2250 |
| 26 |  | > 26 |  | > 2340 |
| 27 |  | > 27 |  | > 2430 |
| 28 |  | > 28 |  | > 2520 |
| 29 |  | > 29 |  | > 2610 |
| 30 |  | > 30 |  | > 2700 |

**Step 2:** **Calculation of modifying points**

Total modifying points = [points of fruits, vegetables & nuts] + [points of fibre] + [points of protein]

For the three food categories, the attribution of points for fruits, vegetables & nuts is modified as follows:

- The points can be scored for fruits, vegetables, nuts and legumes, including coconut, spices, herbs, fungi, seeds, algae and potatoes.
- When the content in fruits, vegetables & nuts is equal to 100%, 8 points are attributed .
- A distinction is made if the food contains concentrated of non-concentrated fruits, vegetables & nuts.

| ***Points*** | **Fruits, Vegetables & nuts (%)** | |
| --- | --- | --- |
|  | Concentrated | Non-concentrated |
| 0 | < 25 | ≤ 40 |
| 1 | ≥ 25 | > 40 |
| 2 | ≥ 43 | > 60 |
| 5 | ≥ 67 | > 80 |
| 8 | = 100 | = 100 |

Protein points: Depending on the average quantity of protein in 100g or 100 ml of food or beverages. A maximum of five points can be awarded.

| Points | Protein (g) per 100g or 100 ml |
| --- | --- |
| 0 | ≤ 1.6 |
| 1 | > 1.6 |
| 2 | ≥ 3.2 |
| 3 | > 4.8 |
| 4 | > 6.4 |
| 5 | > 8.0 |

Fibre points: Fiber points are scored depending on the average quality of dietary fibre in 100g or 100 ml of the food. A maximum five points can be awarded.

| Points | Dietary fibre (g) per 100g or 100 ml |
| --- | --- |
| 0 | ≤0.9 |
| 1 | >0.9 |
| 2 | >1.9 |
| 3 | >2.8 |
| 4 | >3.7 |
| 5 | >4.7 |

For foods from category 1 (beverages), fibre content is not taken into account.

**Step 3: Calculation of the final score**

- Category 1:
- If Total baseline points < 13 points, then NPSC score = Total Baseline points – [points of fruits, vegetables & nuts] - [points of protein]
- If Total baseline points ≥ 13 points and [points of fruits, vegetables & nuts] ≥ 5 then NPSC score = Total Baseline points – [points of fruits, vegetables & nuts] - [points of protein]
- If Total baseline points ≥ 13 points and [points of fruits, vegetables & nuts] < 5 then NPSC score = Total Baseline points – [points of fruits, vegetables & nuts]
- Categories 2 and 3:
- If Total baseline points < 13 points, then NPSC score = Total baseline points – Total modifying points
- If Total baseline points ≥ 13 points and [points of fruits, vegetables & nuts] ≥ 5 then NPSC score = Total baseline points – Total modifying points
- If Total baseline points ≥ 13 points and [points of fruits, vegetables & nuts] < 5 then NPSC score = Total baseline points – [points of fruits, vegetables & nuts] - [points of fibre]

**Reference**

1. (2016) Australia New Zealand Food Standards Code - Schedule 5 - Nutrient profiling scoring method.
