# Supplementary material for: Associations between the nutritional quality of snacks, overall diet quality and adiposity: findings from a nationally representative study of Australian adolescents
Source: Br J Nutr. 2024 Sep 18;132(4):522–31. doi: 10.1017/S0007114524001727 (PMC11499082; doi:10.1017/S0007114524001727)
Supplement: Sisay et al. supplementary material 2 — Sisay et al. supplementary material [file S0007114524001727sup002.docx]

Table 1Characteristics of adolescents between 12-18 years in the National Nutrition and Physical Activity Survey 2011-12 included and excluded from the present study.

|  | Included (784) | | Excluded (151) | | P value^2^ |
| --- | --- | --- | --- | --- | --- |
| Characteristics | Mean | 95 % CI | Mean | 95 % CI |  |
| Age (Years) | 14.8 | 14.6, 14.9 | 14.7 | 14.4, 15.0 | 0.84 |
| Sex |  |  |  |  |  |
| Male | 52.7 | 49.2, 56.2 | 49.0 | 41.1, 56.9 | 0.92 |
| Female | 47.3 | 43.8, 50.8 | 51.0 | 43.1, 58.9 | 0.92 |
| Area-level disadvantage (SEIFA)^1^ quintiles. (%) |  |  |  |  |  |
| Lowest 20% | 15.1 | 12.7, 17.7 | 15.9 | 10.9, 22.6 | 0.56 |
| Second quintile | 18.4 | 15.8, 21.2 | 20.5 | 14.8, 27.7 | 0.42 |
| Third quintile | 22.1 | 19.3, 25.1 | 19.2 | 13.7, 26.3 | 0.08 |
| Fourth quintile | 18.4 | 15.8, 21.2 | 17.9 | 12.6, 24.8 | 0.97 |
| Highest 20% | 26.1 | 23.2, 29.3 | 26.5 | 20.1, 34.1 | 0.88 |
| Waist circumference | 77.1 | 76.3, 77.9 | 80.9 | 67.4, 94.4 | 0.58 |
| WHtR | 0.46 | 0.46,0.47 | 0.46 | 0.03,0.89 | 1.00 |
| Meets physical activity guideline (%) | 7.8 | 6.1, 9.9 | 4.6 | 2.2, 9.4 | 0.79 |
| Snack frequency | 2.3 | 2.2, 2.4 | 2.1 | 2.0, 2.3 | 0.07 |
| ED of meals including beverages | 6.3 | 6.1, 6.4 | 6.1 | 5.8, 6.5 | 0.50 |
| ED of meals without beverages | 6.4 | 6.3, 6.6 | 6.2 | 5.9, 6.6 | 0.32 |
| ED of snacks including beverages | 7.4 | 7.0, 7.8 | 7.3 | 6.5, 8.2 | 0.89 |
| ED of snacks without beverages | 9.5 | 9.1, 10.0 | 9.4 | 8.4, 10.3 | 0.73 |
| NPSC of meals | 4.6 | 4.3, 4.9 | 4.5 | 3.8, 5.3 | 0.92 |
| NPSC of snacks | 10.1 | 9.4, 10.7 | 9.3 | 7.9, 10.8 | 0.35 |
| DGI-CA score | 44.7 | 43.6, 45.7 | 46.3 | 44.0, 48.7 | 0.20 |
| WHtR, Waist-to-height ratio  ED, Energy Density  NPSC, Nutrient Profiling Scoring Criterion, score can range from -13 (most healthy) to 61 (least healthy) ^(1)^  DGI-CA, Dietary Guideline Index – Children and Adolescents ^(2)^  Results are presented as weighted percentages (%) or weighted means (95 % CI)  ^1^Australian Bureau of Statistics Socio-Economic Indexes for Areas. SEIFA quintiles range from one (most disadvantaged) to five (most advantaged)  ^2^ P-value for differences between boys and girls based on F test (continuous variable) or adjusted Wald test (categorical variables) | | | | | |

1. Zealand FSAN (2016) Overview of the Nutrient Profiling Scoring Criterion: Food Standards Australia New Zealand Kingston, Australia.

2. Wilson JE, Blizzard L, Gall SL *et al.* (2019) An age- and sex-specific dietary guidelines index is a valid measure of diet quality in an Australian cohort during youth and adulthood. *Nutr Res* **65**, 43-53.
